# Lyc.—Pneumonia

**Published:** 1881-05

**Authors:** D. Wilson

**Affiliations:** Brook-street, Grosvenor-square


### VETERINARY CASES.

### A SICK COW SAVED BY HOMŒOPATHY.*

*Lycopodium in Pneumonia: Another Proof of its Efficacy.*

SIR.—Your well-known character of independence and love of truth must be my apology for venturing to solicit your aid once more. Five years have elapsed since you first allowed the columns of your influential journal (the London *Morning Advertiser*) to be the medium of my challenge to the London hospitals, when I proposed demonstrating within their wards, then vacant from the want of funds, the great superiority of the homœopathic system of treating the sick, both in a curative and financial point of view. That offer was rejected, although funds to defray every expense were guaranteed, chiefly through the munificence of H. E. Gurney, Esq. Had the experiment of

* Taken from *Monhly Hom. Review.*—1865

three years been successful, there would have been thirty-one endowed beds added to one of our hospitals, and an expenditure of at least £60,000, to £70,000. incurred by a philanthropist. Unless conversant with the facts, one could scarcely believe that philosophy and common sense would, in so noble a cause, have rejected such advantages of unknown magnitude, and which would not have cost the institutions allowing the trial one farthing! Although the London hospitals may for a time have proscribed Truth to obscurity, nevertheless she has not been suppressed, but grows in stregnth, daily, and will shortly triumph over every divice to strangle her. The blessings conferred upon suffering humanity through the genius of Hahnemann are not limited to the human species alone, but are equally effective in the diseases of animals, as the following very interesting case, from among many which I could relate, will prove.

It is necessary that I should explain why I again appeal to the *Morning Advertiser*. When a public journal, whose assertions and teachings are credited, makes an erroneous and unjust statement prejudicial to truth, it is but fair, I submit, on its being corrected, that it should make the *amende honorable* in its very next issue, and thereby disabuse its readers of the errors with which they may have been impressed. This leads me to details. In the weekly paper, the *Field*—circulating, I believe, among farmers and proprietors of live stock—there appeared, on the 27th of last May, a brief communication from "A Subscriber" on the "Homœopathic Treatment of Horses," to which the editor appended a note, wherein he says: "We assert, and have always been ready to prove, that the strength of drugs is not increased by dilution and, trituration, and, in fact, we maintain that the globules sold as homœopathic are completely inert. We defy any one by any means (pathological or chemical) to ascertain the nature of a bottle of globules presented to him without a label, and we are and have long been ready to test their efficacy in this way, for, if it can not be ascertained whether a bottle of globules is composed of arnica, nux vomica, sulphur, phosphorus, or what not, surely there can be no virtue in them." I shall not waste time or space in criticizing this false logic, but I think I am fairly entitled to call upon the editor of the *Field* to fulfil his undertaking, especially if he comprehends what is meant by the "pathological" test for "inert globules." Your readers will now understand my case against the *Field*, wherein I should like to see a little more fairness and no "favor," and which has thought fit to maintain silence on the following communication forwarded on the 24th of June last.

---

*To the Editor of the Field.*

Sir,—As your columns occasionally admit the narration of cures on the homœopathic principle, I hope you may find the following particulars of a very hopeless case of a sick cow successfully so treated of sufficient interest to command a place in your widely read journal. During my visits to a friend suffering from pulmonary hemorrhage, near Iver, Bucks, I was asked if I could advise a neighboring farmer concerning the sudden illness of a valuable Guernsey cow, which had calved on the morning of the previous day (Saturday), June 3, 1865:

Independent of a great fondness for animals, the deep scientific interest and instruction attaching to the study of comparative pathology enlisted, without delay, my services on behalf of the poor animal. On being conducted by a gentleman to the paddock where the cow stood with her calf by her side, I found the farmer, Mr. Goff, Mr. Lamb, (the owner of the cow), the veternary surgeon from Uxbridge, and an old farmer who had seen much illness in animals, all watching the case, the nature of which was a puzzle to all present. I was informed by Mr. Goff —a very intelligent man, who has, in the brief experience of this cow's illness, become, with moderate instruction, a wonderfully expert auscultator—that he left the animal apparently well when he went to church at eleven o'clock, but on his return, between one and two o'clock P. M., he found her unable to walk without a shaking of her limbs, and giving way of her joints, as if she would fall. Believing the illness to be milk fever, aconite and belladonna had been administered frequently, with no beneficial result. The cow could scarcely move a step without appearing as if she would fall. Her injected eyes had a glassy, dull expression of some serious illness. The milk was suppressed, neither had there been for some considerable time any signs of intestinal or urinary function. On applying my ear over the cow's ribs, I soon discovered her disease to be a severe attack of inflammation and congestion of the lungs. Her condition was one of great danger, for which I recommended phosphorus. Mr. Goff tended her all night, scrupulously giving the medicine every two hours. In the early morning (Whit-Monday), I was asked to look at the animal "before she died." On visiting her, in company with another gentleman, I found her lying on her right side under a shed, where she had been for some hours unable to rise. Her neck was stretched out, and on the left side of it there was a large globular swelling, such as may be observed in large fleshy muscles when drawn up in severe cramp. Her breathing was short; her eyelids, when raised by the finger, remained in that position until they slowly and imperfectly recovered their former position. This was a marked proof of exhausted vitality, and the rapidity with which life was ebbing. While making a minute and careful examination into the state of her respiration, the cow gave a distressed moan, as if dying, when I observed a peculiar deep fan-like motion of her nostrils a characteristic symptom for the selection of lycopodium (sometimes called vegetable sulphur) in the treatment of young persons suffering under inflammation of the lungs, and to which I called the attention of the profession and the public in the July number of the *Monthly Homœopathic Review*, 1863. Although years of experience and observation had satisfied me that many of the severe attacks of diseases among animals yielded as rapidly to accurate homœopathic selections administered in an infinitesimal dose, as the like diseases in children, I must confess that I had very little hopes of a favorable result in this extreme case. Twelve globules—yes, twelve globules—of lycopodium, more attenuated or dynamized than the 200th dilution, were dissolved in a quart bottle of cold water, and a table spoonful administered every half hour. I left the apparently dying animal at half past eight on the morning of Whit-Monday, Mr. Goff, Mr. Lamb, and other persons being present, promised to see her when I returned in the afternoon, if she were still alive. As the forenoon advanced, there being no visible improvement and her death being momentarily expected, a messenger was dispatched to Iver, for the butcher to come and kill her. Fortunately, it being Whit-Monday the butchers were absent holiday making. At last a slaughterer was found at Uxbride, but his men were also otherwise engaged, so the poor cow was allowed time for the lycopodium to work upon her disease, and to the astonishment of all who witnessed this apparently hopeless case, the cow rose up and stood firmly on her legs, at a few minutes before 2 P. M. She walked without trembling, and gave most satisfactory evidence that there was neither intestinal nor urinary impediments in her case. I saw her at half-past six in the evening, when she was feeding, and there was scarcely a remnant of the peculiar action of the nostrils to be discovered. One gentleman who observed the symptom remarked that he could never understand what that peculiar action meant, as he had often remarked to his bailiffs and servants when his cattle were dying with pneumonia, "How that beast sneers." This is really a very graphic expression of the symptom as it occurs in animals, and the hint may be of use to future observers. The lycopodium was continued at longer intervals, for although great and marked relief had been afforded to oppressed organic life, it was not to be supposed that a grave lung disease had been thus suddenly cured! In fact, while I write (June 24th) the remnant of the crepitating *rale* characteristic of pneumonia may yet be heard by a capable auscultator in the posterior margin of the left lung. During the progress of the case the left posterior quarter of her udder became hard and tender and suppuration was dreaded. Blood instead of milk came from the teat, still a marked indication for lycopodium, which I ordered to be continued, and the udder threatening also soon disappeared to the delight of Mr. Goff who watched night and day most assiduously this truly marvellous case.

When I state that the last twenty of thirty-five years of my practical experience have been devoted to the laborious study and practice of homœopathy, probably you will consider me no less qualified than yourself to give an opinion on the efficacy of globules, to discover the virtues of which the human organism is a far better and more reliable test than chemistry. I regret that you should have committed the error of imagining that "the globules sold as homœopathic are completely inert," because chemical science has hitherto failed to analyse them. Where is the chemist to be found who can discover the least difference between gum water and the deadly poison of venomous serpents? If you are willing to submit to the physiological test I shall have much pleasure in taking you in hand, then you will be enabled to speak practically and positively. Allow me to remark that there can be no such illogical absurdities as homœopathic globules "on sale." Globules and other forms of medicinal preparation can only be called homœopathic when they meet with their corresponding affinities in the symptoms of disease. The totally different terms, homœopathic and infinitesimal, are constantly being confounded by people who should know better. Similarity between the symptoms of the disease and those of the remedy, and not the magnitude of the dose, alone constitute homœopathicity. The dose, to be efficient, however, according to the homœopathic principle, as correct experience proves, ought to be administered in an infinitesimal state of preparation. To heal the sick a dose much less than that which was required to make healthy people ill is amply sufficient for all curative purposes. When a material dose is used such may act and often does so on the allopathic principle even in animals, The susceptibility of the diseased organism, in man and animals, to medicinal impressions, surpasses all belief. Hahnemann has never said, that by "dilution and trituration" medicines were increased in strength, as you seem to imply, according to the common definition of that word. His translators have done him great injustice on many occasions, and in none more so than in the translation of the word (kraft) by strength. Kraft in medicine according to Hahnemann's use of the word means virtue or efficacy and not brute strength. In answer to your Natal correspondent of last week, asking for information in regard to the sore mouths in sheep passing into "blue tongues" when they fall down, kick and die; I should recommend him to study out of a reliable Materia Medica the action of arsenic, muriatic acid, digitalis, and sabadilla. Among the first two or three remedies he will propably find more corresponding to the whole disorder of the sheep than he has yet observed. I should like to know the effect of your calomel prescription for the Cochin-Vertigo.—June 24th, 1865.

I should have thought after the display of so much confidence that no editor of sufficient courage and desirous to elicit truth, would have shrunk from my challenge—the test proposed by himself—in which I would promise not to poison him.

I am, sir, yours faithfully,

D. Wilson, M. D.

Brook-street, Grosvenor-square,

July 30, 1865.

